# Care pathways, service access, and psychosocial support needs in children and adolescents with autism spectrum disorder: a narrative review

**DOI:** 10.3389/fpubh.2026.1840171

**Published:** 2026-09-11

**Authors:** Xugao Han, Lingyan Weng, Houxi Xu

**Affiliations:** 1 School of Medical Humanities, Nanjing University of Chinese Medicine, Nanjing, China; 2 School of Tourism Management, Nanjing Institute of Tourism and Hospitality, Nanjing, China; 3 Key Laboratory of Acupuncture and Medicine Research of Ministry of Education, Nanjing University of Chinese Medicine, Nanjing, China

**Keywords:** autism spectrum disorder, care pathways, children, service access, transition

## Abstract

Autism spectrum disorder (ASD) is a heterogeneous neurodevelopmental condition that often requires long-term support across healthcare, education, and social care systems. Despite increasing recognition, many children and adolescents continue to experience delayed identification, fragmented care pathways, and unequal access to services. This narrative review synthesizes recent evidence on care pathways, service access, and psychosocial support needs in children and adolescents with ASD, drawing on literature published from 2020 onward, with emphasis on recognition, barriers to diagnosis and post-diagnostic support, service access, family burden, school participation, and transition-related needs. Recent evidence indicates that ASD-related care remains uneven across regions and systems. Delays in referral and assessment, workforce shortages, long waiting times, socioeconomic and geographic disparities, and poor coordination across healthcare, education, and social care remain major barriers to timely support. These structural constraints increase caregiver burden, complicate school participation, and weaken continuity of care during adolescence and transition to adulthood. Families frequently report difficulties navigating complex systems, while schools function as both a major site of support and a major source of unmet need. Improving outcomes requires earlier recognition, more equitable access to multidisciplinary services, stronger family and school support, and better coordination across healthcare, education, and social care sectors. A developmental and systems-oriented approach is likely to better address the changing needs of children and adolescents with ASD.

## 1 Introduction

Autism spectrum disorder (ASD) is a lifelong neurodevelopmental condition characterized by differences in social communication and interaction, together with restricted or repetitive behaviors, interests, or sensory responses (1–4). Although the core diagnostic criteria are well established, ASD is highly heterogeneous in clinical presentation and support needs. Children and adolescents with ASD differ substantially in language development, cognitive ability, adaptive functioning, sensory processing, emotional and behavioral regulation, and co-occurring psychiatric or medical conditions (1–6). This heterogeneity has important implications for care, because the needs of autistic children and adolescents rarely fit a single clinical pathway, intervention model, or service setting.

The public health relevance of ASD has become increasingly apparent. Recent surveillance data from the United States estimated that approximately 1 in 31 children aged 8 years were identified with ASD in 2022, while the Global Burden of Disease Study 2021 estimated that approximately 1 in 127 people worldwide were autistic (1, 2). These estimates indicate not only the frequency of ASD, but also the scale of need for developmental surveillance, diagnostic assessment, family guidance, educational accommodations, psychosocial support, and long-term transition planning across childhood and adolescence (1, 2, 7, 8). In this sense, ASD should be understood not only as an individual developmental diagnosis, but also as a major child-health, educational, and service-planning concern.

Despite increasing recognition, many children and adolescents with ASD continue to encounter delayed identification, prolonged waits for specialist assessment, fragmented post-diagnostic support, and unequal access to therapy, educational accommodations, and community-based services (5, 7–15). These barriers are often most pronounced among children from socioeconomically disadvantaged families, racially or linguistically minoritized groups, rural or under-resourced areas, and those whose clinical presentations do not match traditional expectations of autism (9, 10, 12–18). As a result, outcomes are shaped not only by the characteristics of the child, but also by how quickly systems recognize developmental concerns, how effectively families are connected to diagnostic and post-diagnostic services, and how well healthcare, education, and social care sectors coordinate support over time.

These interconnected challenges highlight why care pathways, service access, and psychosocial support needs should be examined together rather than as isolated topics. Care pathways describe the sequence through which children and families move from first concerns to referral, diagnostic assessment, post-diagnostic planning, school-based support, and transition to adult-oriented services. Service access determines whether these pathway stages are timely, affordable, understandable, and practically usable for families. Psychosocial support needs reflect the downstream consequences of pathway quality, including caregiver burden, school participation, mental health needs, family stress, and continuity of care during adolescence (19–31). When pathways are delayed or poorly coordinated, families often become default care coordinators, schools may struggle to implement appropriate supports, and adolescents may face discontinuity as they move from child-centered to adult-oriented systems.

Although existing literature has addressed several components of ASD care, including early identification, diagnostic disparities, intervention access, caregiver burden, school participation, and transition planning, these domains have largely been examined as separate research lines rather than as interconnected stages of a developmental care pathway. Less attention has been given to how these domains are connected across childhood and adolescence. This gap is important because fragmentation in ASD care rarely occurs at a single point. Instead, difficulties may accumulate across sequential stages, from delayed recognition and diagnostic assessment to inconsistent post-diagnostic follow-up, inadequate school support, and poorly coordinated transition planning. A pathway-oriented synthesis is therefore needed to clarify how service structures shape psychosocial outcomes and to identify points at which care systems can become more equitable, coordinated, and responsive (6, 8, 28, 31, 32).

This review was guided by the following research question: What does recent evidence indicate about care pathway organization, barriers to service access, and psychosocial support needs among children and adolescents with ASD, and what are the implications for service design across healthcare, education, and social care systems? To address this question, this narrative review synthesizes recent evidence on care pathways, service access, and psychosocial support needs in children and adolescents with ASD. The review focuses on recognition and early identification, diagnostic and post-diagnostic pathways, barriers to service access, family burden, school participation, and continuity of care during the transition to adulthood. It does not address symptom biology or pharmacological treatment. Instead, it examines how healthcare, education, and family support systems shape the everyday experiences and longer-term outcomes of children and adolescents with ASD. By integrating these domains within a developmental and systems-oriented framework, this review aims to examine why timely diagnosis alone may not be sufficient and how coordinated, family-centered, and developmentally continuous support across healthcare, education, and social care systems can be strengthened.

## 2 Methods

### 2.1 Study design

This article was designed as a narrative review to provide an integrative synthesis of recent evidence on care pathways, service access, and psychosocial support needs in children and adolescents with autism spectrum disorder (ASD). Particular attention was given to how these domains interact across a developmental continuum from early childhood to adolescence. A narrative review approach was considered appropriate because the purpose of this article was not to estimate pooled effects or conduct a meta-analysis, but rather to integrate evidence from heterogeneous sources and interpret how healthcare, education, and family support systems shape ASD-related care. Narrative reviews are particularly useful when the aim is to synthesize diverse evidence, develop conceptual understanding, and provide interpretive analysis of broad or complex topics rather than produce pooled quantitative estimates. Consistent with this aim, the review prioritized thematic interpretation and conceptual synthesis over formal effect estimation. Nevertheless, the search, selection, and synthesis procedures were reported as explicitly as possible to improve methodological transparency (33, 34).

### 2.2 Information sources and search strategy

Literature was identified primarily through PubMed searches conducted between January and March 2026. The search covered publications from January 2020 to March 2026. These 6 years were selected to capture recent evidence reflecting contemporary ASD service contexts, including changes in care delivery associated with and following the COVID-19 pandemic. Additional sources were identified through guideline databases, public-health surveillance reports, and reference list screening of relevant articles, particularly for foundational diagnostic references and recent epidemiological surveillance reports.

The PubMed search strategy combined terms describing the target population with terms corresponding to the major thematic axes of this review. The full search string was as follows:

(“autism spectrum disorder”[Title/Abstract] OR “autistic”[Title/Abstract]) AND (“child*”[Title/Abstract] OR “adolescen*”[Title/Abstract]) AND (“care pathway*”[Title/Abstract] OR “service access”[Title/Abstract] OR “service use”[Title/Abstract] OR “unmet need*”[Title/Abstract] OR “caregiver”[Title/Abstract] OR “family burden”[Title/Abstract] OR “school participation”[Title/Abstract] OR “transition”[Title/Abstract] OR “health disparities”[Title/Abstract] OR “health inequit*”[Title/Abstract]) AND (“2020/01/01”[Date - Publication]: “2026/03/31”[Date - Publication]). This primary PubMed search yielded 1,330 records. Only English-language sources were considered eligible for inclusion.

### 2.3 Inclusion and exclusion criteria

Sources were considered eligible if they were published between January 2020 and March 2026, with limited exceptions made for foundational diagnostic references, such as DSM-5-TR, that were retained for conceptual continuity. Eligible sources focused on children and/or adolescents aged 0–18 years with ASD, or on transition-aged youth when adolescent experiences or the transition from adolescence to adulthood were explicitly examined. To be included, sources also had to address at least one of the six thematic axes of the review: recognition and early identification, diagnostic and post-diagnostic pathways, service access and unmet needs, family burden and caregiver support, school participation, or transition to adulthood. Peer-reviewed empirical studies, qualitative studies, scoping reviews, systematic reviews, clinical practice guidelines, and population-based surveillance reports were considered eligible. Only English-language sources were included.

Sources were excluded if they focused exclusively on adult ASD populations without adolescent or developmental relevance, addressed only neurobiological mechanisms, genetics, biomarker discovery, or pharmacological treatment, or did not contribute directly to the review’s focus on care pathways, service access, or psychosocial support needs. Non-peer-reviewed sources, including editorials, opinion pieces, news commentary, and non-indexed preprints, were excluded unless they provided essential surveillance or guideline information not available elsewhere. Sources unavailable in full text or in English were also excluded.

Survey-based studies, caregiver-reported studies, and secondary analyses of existing datasets were eligible when they directly addressed one or more of the review’s thematic domains, including diagnostic pathways, service access, unmet needs, caregiver burden, school participation, or transition-related support. These study designs were not excluded *a priori*. However, because this was a narrative review rather than an exhaustive systematic review of all service-use literature, selection within these categories was guided by relevance to the developmental care-pathway framework, recency, clarity of population and methods, and contribution to understanding service organization or psychosocial support needs. COVID-19-related studies were included when they examined disruption, adaptation, or recovery of ASD-related diagnostic, therapeutic, educational, or family-support services. Studies focused primarily on general pandemic stress, broad mental health effects, or family experiences without a clear link to ASD care pathways, service access, or psychosocial support were excluded from the main synthesis.

### 2.4 Screening and selection

Records identified through the primary PubMed search were screened in two stages. First, titles and abstracts were reviewed against the predefined inclusion and exclusion criteria. Records were excluded at this stage if they clearly focused on adult-only populations without developmental relevance, neurobiological mechanisms, genetics, biomarker discovery, pharmacological treatment, or unrelated clinical topics. Second, records considered potentially relevant after title and abstract screening were assessed in full text to determine whether they contributed substantively to one or more of the review’s thematic axes.

Screening was performed by the first author according to the predefined criteria, and the final list of sources used for synthesis was reviewed by all co-authors. Because this review used a narrative and interpretive synthesis approach, study selection emphasized thematic relevance, recency, sufficient methodological reporting, and contribution to the developmental and systems-of-care framework adopted in the review. In this context, methodological transparency was not assessed using a formal scoring instrument. Instead, it referred to whether a source provided sufficient information about its study aim, population, setting, data source, data collection or synthesis methods, key outcomes, and limitations to allow its relevance and contribution to the review question to be interpreted. This criterion was used to inform judgment during article selection and synthesis, particularly when multiple studies addressed similar topics, but it was not applied as a numerical quality score or independent exclusion threshold. Any uncertainties about article relevance or inclusion were resolved through discussion and consensus among the authors. Because duplicate independent screening was not conducted, no formal inter-rater agreement statistic was calculated.

Following full-text assessment and the incorporation of additional foundational sources identified through supplementary searches, eligible sources were included to inform the thematic synthesis. When multiple studies addressed similar questions, priority was given to recent, sufficiently reported, and contextually informative work, including studies involving under-represented populations or low- and middle-income settings, in order to support a balanced and equity-sensitive synthesis.

### 2.5 Synthesis approach

The included sources were organized according to the six thematic domains of the review and synthesized narratively. Within each domain, evidence was examined to identify recurring patterns, points of convergence, areas of disagreement or uncertainty, and gaps relevant to service design. The synthesis was developmentally informed, with attention to how needs and system responses evolve from early childhood through adolescence, and equity-sensitive, with attention to differences across geographic, socioeconomic, linguistic, and ethnic groups. The main evidence domains and representative sources are summarized in Table 1, and a more detailed evidence map is provided in Supplementary Table 1.

**Table 1:** Summary of evidence domains and representative sources included in the narrative synthesis.

Evidence domain	Main source types	Representative sources	Main contribution to the synthesis
Public health relevance and heterogeneity of need	Surveillance report, global burden study, diagnostic reference, public-health review, health-services review	Shaw et al. (1); Santomauro et al. (2); Ostrowski et al. (3); American Psychiatric Association (4); Divan et al. (7); Malik-Soni et al. (8)	Establishes ASD as a major public-health and service-planning issue characterized by increasing identification, heterogeneous support needs, co-occurring conditions, and long-term care requirements across childhood and adolescence.
Recognition, early identification, and barriers to diagnosis	Diagnostic pathway studies, disparity studies, systematic/scoping reviews, and telehealth-related studies	Zavaleta-Ramírez et al. (5); Aylward et al. (9); Weiss et al. (10); Bent et al. (11); Miller et al. (12); Onovbiona et al. (13); Wagner et al. (14); Lockwood Estrin et al. (18); Kavanaugh et al. (35); Wallis et al. (40)	Shows that diagnostic timing is shaped not only by child characteristics, but also by referral pathways, service availability, race and ethnicity, language, gender-related presentation, family resources, geography, and access to trained providers.
Diagnostic and post-diagnostic pathways	Integrated care pathway review, quality-improvement study, family navigation studies, digital health, and service navigation studies	Fulceri et al. (6); Malik-Soni et al. (8); Landau-Taylor et al. (19); Burke et al. (23, 24); Mazurek et al. (25); Li et al. (26)	Supports the shift from a diagnosis-centered model to a pathway-centered model in which diagnosis functions as an entry point to coordinated post-diagnostic support, family navigation, school linkage, and developmental follow-up.
Service access and unmet needs	Cross-national studies, caregiver-perspective studies, survey-based studies, geographic access studies, equity-focused reviews, universal health coverage reviews	Divan et al. (7); Malik-Soni et al. (8); Casale et al. (15); Lindsay et al. (16); Hantman et al. (17); Micai et al. (20); Saleh et al. (21); Pillay et al. (22); Sultan (27); Song et al. (28); Binte Mohd Ikhsan et al. (41); Srinivasan et al. (43)	Demonstrates persistent barriers to service access, including waiting times, costs, workforce shortages, poor coordination, geographic disparities, language barriers, migration-related barriers, limited service awareness, and unmet healthcare and family support needs.
Family burden and caregiver support needs	Cross-sectional studies, qualitative studies, caregiver-perspective studies, scoping/systematic reviews, caregiver support, and parent-focused intervention studies	Desquenne Godfrey et al. (29); Bradshaw et al. (32); Lockwood Estrin et al. (42); Emmanuel et al. (44); Herrero et al. (45); van Niekerk et al. (46); Asahar et al. (47); Robeson et al. (48); Appah et al. (49); D’Arcy et al. (50); Bar et al. (51); Davy et al. (52, 53); Postma et al. (54); Reichow et al. (55)	Indicates that caregiver burden reflects both child-related support needs and system-level fragmentation, including financial strain, psychological distress, reduced caregiver participation, reduced quality of life, and the need for psychoeducation, peer support, navigation assistance, and caregiver mental health support.
School participation, psychosocial functioning, and outcome perspectives	School participation studies, environmental participation studies, stakeholder research, educational placement studies, inclusive education studies, neurodiversity-affirming outcome framework	Zhao et al. (30); Hughes et al. (36, 37); Waddington et al. (38); Walton et al. (39); Li et al. (54); Simpson and Adams (57); Golos et al. (58); Hasson et al. (59); Pellicano and Heyworth (62)	Frames school participation as a health-relevant and psychosocial outcome shaped by environmental fit, accommodations, inclusive practice, sensory and social context, and coordination between healthcare and education systems. It also broadens outcome evaluation beyond symptom reduction toward meaningful participation, autonomy, belonging, and autistic flourishing.
Adolescence, continuity of care, and transition to adulthood	Population-based adolescent studies, mental health service-use studies, qualitative transition studies, transition-focused survey studies	Song et al. (28); Davis et al. (31); Hughes et al. (36, 37); Ames et al. (60); Zhang et al. (61)	Highlights adolescence and transition to adulthood as periods of increased vulnerability, when service discontinuity, mental health needs, school-to-adult system gaps, eligibility changes, and weak coordination may intensify existing inequities.
COVID-19-related temporal context and service disruption	COVID-19 cohort/survey studies, service recovery analyses, post-diagnostic service-access studies, telehealth uptake studies	Landau-Taylor et al. (19); Wallis et al. (40); White et al. (63); Tsai and Bhat (64)	Provides contextual evidence showing that the COVID-19 pandemic disrupted diagnostic, therapeutic, educational, and family-support services; amplified pre-existing care pathway weaknesses; increased caregiver burden; accelerated telehealth use; and revealed demographic inequities in service disruption and recovery.

### 2.6 Quality appraisal

Consistent with the methodological framework of a narrative review, no formal quality appraisal instrument, such as AMSTAR-2, ROBIS, CASP, PEDro, or the NIH Quality Assessment Tools, was applied. This decision reflected the heterogeneity of the included evidence, which comprised surveillance reports, clinical guidelines, empirical studies, qualitative studies, caregiver-perspective studies, scoping reviews, systematic reviews, and service-access studies. However, study quality and methodological limitations were considered during synthesis.

The included sources were not treated as equivalent in evidential weight. During synthesis, attention was given to study design, population and sample characteristics, setting, data source, outcome focus, clarity of reporting, and limitations acknowledged by the original authors. Greater interpretive weight was generally given to population-based surveillance reports, systematic or scoping reviews, clinical guidelines, and empirical studies with clearly described methods and populations when they directly addressed the review question. Qualitative studies, caregiver-perspective studies, and single-site studies were used primarily to contextualize lived experiences, barriers to service navigation, family burden, and psychosocial support needs rather than to estimate prevalence, service effects, or generalizable associations. Findings from small-sample or highly context-specific studies were interpreted cautiously and were integrated mainly when they illustrated mechanisms, barriers, or contextual variation that were consistent with broader evidence. Where possible, conclusions were based on convergence across multiple sources, study designs, and service contexts rather than on isolated findings from a single study.

## 3 Results: evidence mapping and thematic synthesis

### 3.1 Characteristics of the included evidence

The evidence base used for the thematic synthesis comprised 62 ASD-related sources. Methodological references cited to support the narrative review approach were not included in these descriptive statistics. For descriptive purposes, each source was assigned to one broad source-type category according to its primary design or function in the synthesis. Empirical quantitative, mixed-methods, survey-based, service-access, pathway, and implementation studies accounted for 32 sources (51.6%). Reviews and evidence syntheses, including systematic reviews, scoping reviews, reviews of reviews, clinical reviews, and qualitative meta-syntheses, accounted for 16 sources (25.8%). Qualitative or caregiver-perspective primary studies accounted for 8 sources (12.9%). The remaining 6 sources (9.7%) comprised surveillance or global burden reports, diagnostic references, policy or perspective articles, stakeholder research agendas, and conceptual/theoretical articles.

The geographic and contextual distribution of the evidence was uneven. Based on the primary country or setting reported in the evidence map, 22 sources (35.5%) were primarily based in the United States. Other high-income English-speaking settings accounted for 3 sources (4.8%), including Australia and Canada. Other high-income or regional settings, including Italy, Saudi Arabia, Israel, and European service contexts, accounted for 6 sources (9.7%). Low- and middle-income or non-Western country-specific settings accounted for 6 sources (9.7%), including China, India, South Africa, Ghana, and Malaysia. The remaining 25 sources (40.3%) were global, international, conceptual, review-based, or not tied to a single country or service setting.

For the purpose of summarizing the thematic structure of the evidence base, each source was also assigned to one primary thematic contribution, although many sources informed more than one domain. Public-health relevance and foundational diagnostic evidence accounted for 4 sources (6.5%). Recognition, early identification, diagnostic pathways, post-diagnostic support, and family navigation accounted for 15 sources (24.2%). Service access, unmet needs, equity, and COVID-19-related service disruption accounted for 15 sources (24.2%). Family burden, caregiver support, and caregiver quality of life accounted for 15 sources (24.2%). School participation, inclusive education, psychosocial functioning, and neurodiversity-affirming outcome perspectives accounted for 10 sources (16.1%). Adolescence, continuity of care, and transition to adulthood accounted for 3 sources (4.8%).

Sample sizes were not uniformly reported across all included sources because the evidence base included reviews, surveillance reports, diagnostic references, conceptual articles, qualitative studies, survey-based studies, and empirical service-access studies. Accordingly, the synthesis emphasized the type of evidence, population and setting, thematic contribution, and convergence of findings across sources rather than pooled sample-size estimates. Detailed study characteristics are provided in Supplementary Table 1.

### 3.2 Thematic organization of the evidence

The literature used for this narrative synthesis was methodologically heterogeneous and covered multiple levels of ASD-related care. This heterogeneity reflects the multisectoral nature of ASD care, in which healthcare, education, family support, and social care systems interact across development. Accordingly, the evidence was organized into interconnected domains corresponding to the developmental and systems-oriented framework of this review: public health relevance and heterogeneity of need, recognition and early identification, diagnostic and post-diagnostic pathways, service access and unmet needs, family burden and caregiver support needs, school participation and psychosocial functioning, and adolescence, continuity of care, and transition to adulthood.

These domains were not treated as isolated categories. Early recognition influences diagnostic timing; diagnostic assessment shapes access to post-diagnostic support; service availability affects family burden and school participation; and gaps in childhood services may accumulate during adolescence and transition to adulthood. Table 1 provides a concise summary of these evidence domains and relevant cross-cutting contextual evidence, while Supplementary Table 1 presents the detailed evidence map of sources used for the thematic synthesis.

## 4 Public health relevance and heterogeneity of need

The clinical and service implications of ASD begin with its prevalence and heterogeneity. Surveillance and global burden estimates consistently show that ASD is common, but the significance of prevalence extends beyond case counts. Higher identification rates imply a growing number of children who may require developmental assessment, communication support, parent guidance, school accommodations, and transition planning. At the same time, children and adolescents with ASD do not constitute a homogeneous service population. Some present with marked communication and adaptive difficulties in early childhood, whereas others are recognized later, often in the context of increasing school demands, co-occurring anxiety, or subtler but impairing social-communication differences (2, 5, 6, 9, 18, 35).

Service planning is further complicated by the high frequency of co-occurring conditions. Population-based adolescent studies have shown that ADHD, anxiety, and other health or educational needs commonly coexist with ASD, and that patterns of identification and service use vary by intellectual disability status, demographic factors, and locality (28, 31, 36, 37). This means that healthcare and educational systems must frequently respond to multiple overlapping needs rather than to ASD alone. Autism care is therefore rarely confined to a single specialty or a single clinic; instead, it unfolds across pediatric services, developmental-behavioral assessment, school systems, rehabilitation providers, community organizations, and family support networks (5, 6, 8, 19–23, 26).

Another implication of heterogeneity is that successful care cannot be defined solely by diagnostic timing or symptom reduction. Support priorities differ across clinicians, parents, and autistic communities. Participatory and community-based research suggests that improving quality of life, reducing harmful behaviors, and modifying environments to better support the child are often regarded as more meaningful priorities than attempting to normalize autistic traits or impose narrow performance-based goals. This perspective aligns with a broader shift toward developmental and participation-oriented models of care (32, 38, 39).

## 5 Recognition and early identification

Although autistic characteristics may be detectable in early childhood, recognition is often delayed or unevenly distributed (1, 5, 9–14, 16, 18). In practice, first concerns frequently emerge outside specialist settings. Parents may notice reduced social reciprocity, delayed communication, unusual sensory responses, rigid play, or difficulty adapting to change. Teachers may observe social difficulties, classroom distress, atypical peer interaction, or behavioral dysregulation that becomes more visible as academic and social demands intensify. The pathway from concern to referral, therefore, depends heavily on non-specialist adults whose experience, knowledge, and access to support may vary substantially.

Clinical guidance emphasizes the importance of timely recognition, referral, and multidisciplinary assessment (5). However, implementation in real-world settings remains inconsistent. Aylward and colleagues described persistent racial, ethnic, and sociodemographic disparities in ASD diagnosis (9). More recent equity-oriented reviews further indicate that language barriers, ethnicity, reimbursement structures, and limited access to trained providers or interpreters can contribute to diagnostic delay and reduced access to appropriate psychosocial support (10, 12, 13, 15, 16). These findings suggest that delayed identification is not simply a matter of symptom visibility; it is also shaped by structural inequality.

Cultural and linguistic context may also shape recognition before a child reaches specialist assessment. The included equity-focused literature suggests that language and cultural barriers, family and community understandings of autism, stigma, stereotypes, and the quality of family–provider communication may influence whether developmental concerns are recognized, discussed, referred, and followed through to diagnostic evaluation (10, 13, 15, 16). These influences should not be interpreted as fixed characteristics of particular cultural groups; rather, they interact with structural factors such as health literacy, access to culturally responsive services, and the availability of appropriate communication support.

Recognition can also be delayed when autism is identified later in school age or adolescence. Clinical reports from ethnically diverse evaluation settings show that a meaningful minority of children receive an initial autism diagnosis after age 7, often with co-occurring language or anxiety disorders and without prior parental concern specifically focused on autism (18, 35). Such cases remind clinicians that school-age and adolescent diagnosis is not rare and that late recognition may reflect the interaction of social demands, co-occurring conditions, and prior system-level barriers.

From a health-services perspective, early identification matters because it determines whether children and families can access developmental guidance, educational planning, and interventions while demands are still evolving (1, 5, 7, 11). But improving early recognition requires more than better screening tools. It also requires culturally and linguistically responsive pathways, provider training, and referral systems that are accessible to families with different levels of health literacy and system familiarity (10, 13–16, 25, 40).

## 6 Diagnostic and post-diagnostic pathways

ASD diagnosis is ideally based on a multidisciplinary, developmentally informed assessment (5). Clinical guidelines recommend integrating developmental history, direct observation, cognitive and language information, adaptive functioning, and co-occurring psychiatric or medical issues when reaching diagnostic conclusions and planning support. In theory, such an approach allows clinicians to move beyond labeling and toward functional, individualized recommendations.

In practice, however, the pathway from first concern to diagnosis is frequently prolonged, fragmented, and difficult for families to navigate (6, 11, 14, 19–23, 26). Families may move between primary care, community services, educational referrals, and specialist centers before reaching definitive assessment. Delays can arise because of long waiting lists, regional shortages of developmental specialists, or insufficient clarity about who is responsible for referral and follow-up (17, 19, 22, 24). Geographic analyses in the United States have documented substantial disparities in the availability of general and specialized pediatricians, including areas with low provider access but relatively high estimated neurodevelopmental disorder prevalence (17). Such structural imbalances directly shape how quickly children enter care pathways.

The period after diagnosis is often as challenging as the period before it. Landau-Taylor and colleagues reported that many children diagnosed during the COVID-19 period did not receive all recommended services and that developmental follow-up was inconsistent (19). This reflects a recurrent problem in autism care: diagnosis can function as an administrative endpoint rather than a coordinated entry point into support. Families may leave assessment appointments with recommendations but little practical help in translating those recommendations into therapy, school services, community supports, or realistic next steps (6, 8, 20–22, 26, 28).

Recent work on family navigation addresses this gap. A scoping review by Li et al. found that family navigation programs can accelerate movement from positive screening to diagnosis and from diagnosis to intervention while also supporting caregiver outcomes (26). Navigation activities included service coordination, psychosocial support, health education, advocacy, and logistical assistance. Additional studies have shown that digital navigation tools and integrated models linking primary care training with family navigation may improve caregiver experience and provider confidence, especially in underserved communities (6, 14, 24, 25). These findings suggest that effective care pathways require more than diagnostic capacity. They require coordination capacity.

This shift from “diagnosis-centered” to “pathway-centered” thinking is essential. In a pathway-centered model, recognition, referral, assessment, post-diagnostic planning, school linkage, family support, and follow-up are treated as connected stages rather than disconnected episodes. Such a model is particularly relevant for health services research, because it frames ASD not only as a clinical entity but also as a care-delivery challenge (6, 8).

## 7 Service access and unmet needs

Access to ASD-related services remains one of the most persistent challenges identified in recent literature (24–26). Across countries and systems, families report barriers related to waiting times, cost, workforce shortages, service fragmentation, weak communication across sectors, and difficulty understanding what services exist or how to obtain them (8, 15, 20–23, 26, 28, 41, 42). Survey-based evidence has similarly shown that caregivers of children and youth with ASD report substantial unmet needs in healthcare access and family support services, including difficulties obtaining therapies, navigating available resources, and securing practical guidance for families (43). These barriers are not trivial inconveniences. They shape whether a child receives communication support, behavioral guidance, educational accommodations, family psychoeducation, or continuity of care over time.

Recent studies provide concrete examples of these barriers. In Italy, Micai et al. found that carers of children and adults with ASD often relied on a mixture of public and privately financed services, with substantial caregiving time commitments and significant direct cost burdens (20). In Saudi Arabia, Saleh et al. reported variability in parental satisfaction with services, indicating that availability alone does not guarantee responsiveness or perceived usefulness (21). In South Africa, qualitative work captured caregivers’ experiences of long delays and profound frustration while waiting for school placements and autism-related services, with families describing the financial, emotional, and time-related “costs of waiting” (22).

Service access is especially difficult for low-resourced and minoritized families (12, 13, 15, 16, 23, 44). Burke and colleagues found that parents from low-resourced communities identified limited service awareness, difficulty understanding diagnosis, and a need for advocacy and peer support as major barriers. Other studies have shown that Latinx families may face health literacy barriers even within integrated healthcare systems, and that racism, poor quality interactions, language barriers, and stereotypes continue to shape healthcare experiences for children with ASD and their caregivers (13, 16). Equity concerns also extend to telehealth. Although telehealth has the potential to improve reach, disparities in telehealth uptake by preferred family language have been documented in developmental-behavioral pediatric settings (14, 40).

**Figure 1:**
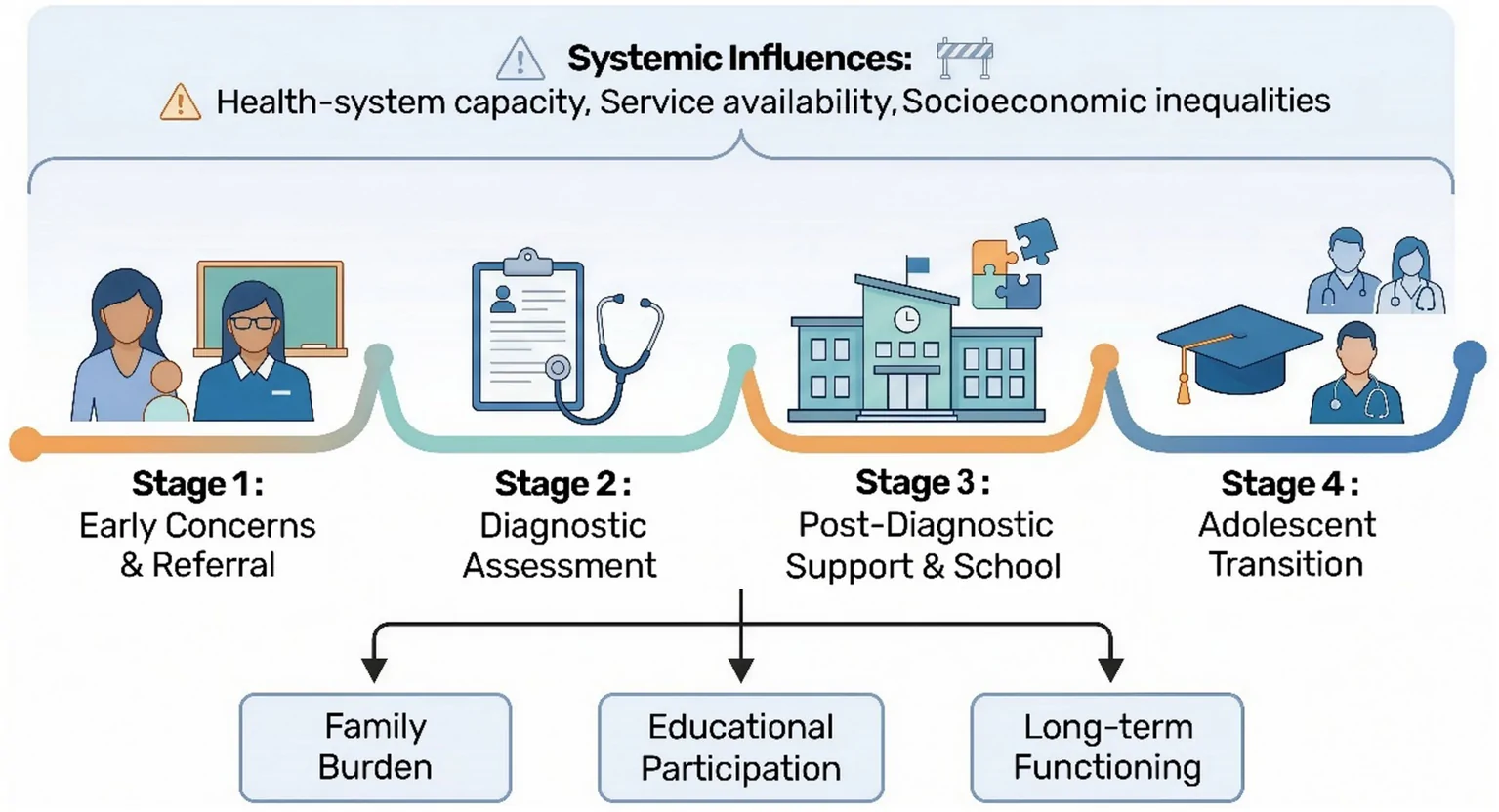
Conceptual framework of ASD care pathways and psychosocial support needs across development. This horizontal timeline illustrates the progression from early concerns and referral (Stage 1) through diagnostic assessment, post-diagnostic support, and adolescent transition (Stage 4). An overarching bracket highlights how health-system capacity, service availability, and socioeconomic inequalities systemically influence all stages of care. These structural factors ultimately shape major downstream outcomes (bottom boxes), including family burden, educational participation, and long-term functioning.

These patterns support a broader understanding of unmet need. In ASD, unmet need is not simply the absence of therapy hours (8, 21–23, 28, 41). It includes the absence of interpretable information, poor care coordination, long travel or waiting burdens, inconsistent follow-up, and a mismatch between family priorities and service models. It is therefore possible for a service system to appear active on paper while still being experienced as inaccessible or ineffective by families (Figure 1).

**Figure 2:**
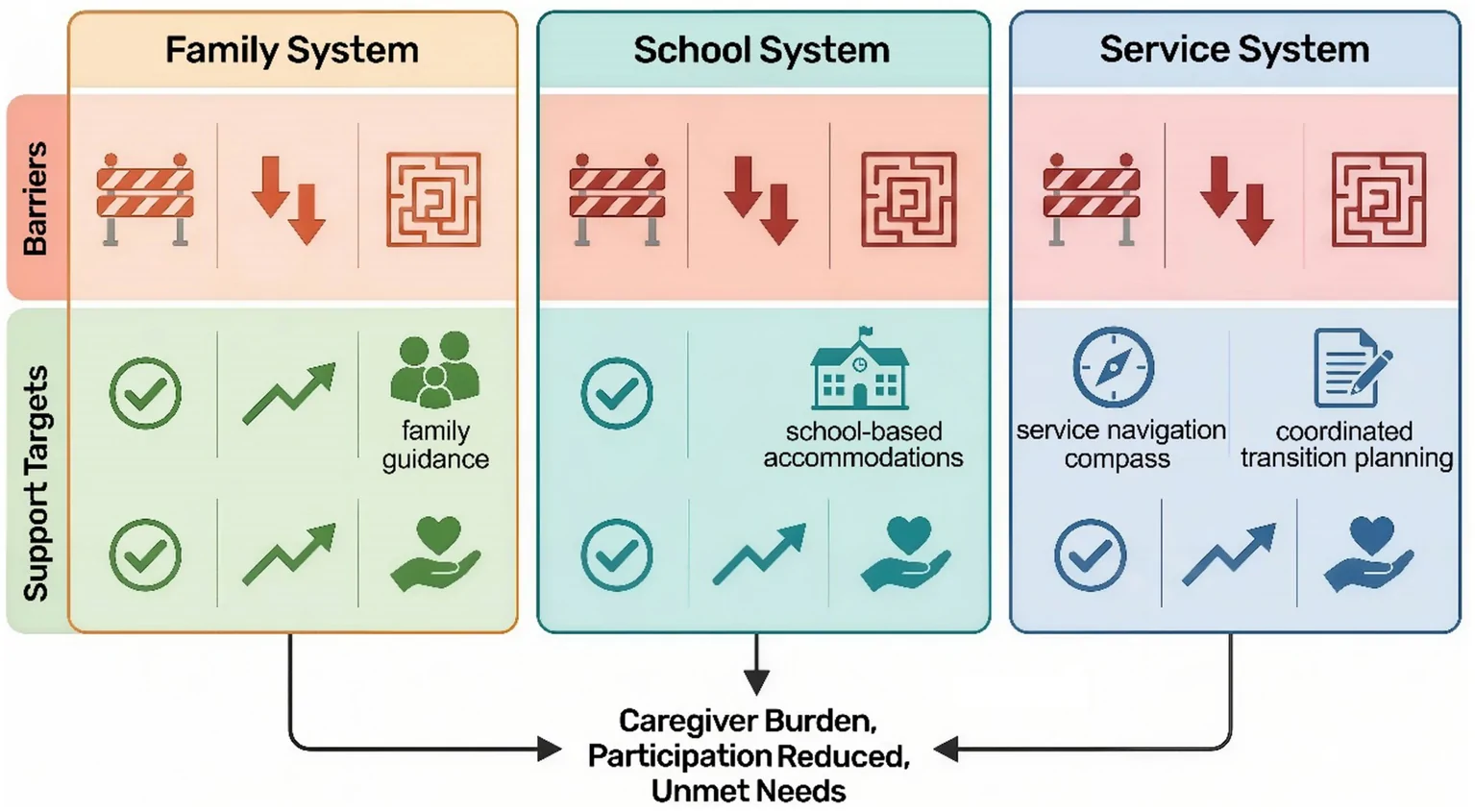
Multilevel barriers and support targets for children and adolescents with ASD across family, school, and service systems. The figure summarizes how barriers and potential support targets operate across three interconnected systems: the family system, school system, and service system. In the upper “Barriers” row, roadblock icons represent structural or practical barriers to access, downward arrows indicate reduced in access, continuity, or participation, and maze icons represent complex navigation demands across systems. In the lower “Support Targets” row, checkmarks indicate priority points for feasible intervention, upward arrows indicate strengthening of support or improved participation, and hand/heart icons represent psychosocial, relational, and family-centered support. The family-system panel highlights family guidance and caregiver support; the school-system panel highlights school-based accommodations and participation support; and the service-system panel highlights service navigation and coordinated transition planning. The converging arrows at the bottom indicate that barriers and supports across these systems jointly shape caregiver burden, participation barriers, and unmet needs.

This perspective is increasingly recognized in the literature. Sultan argued that equitable and sustainable autism care requires not only more services but also better distribution, provider training, telehealth infrastructure, community-based support, and targeted financial assistance (27). Divan et al., in their review of universal health coverage for young children with ASD in low- and middle-income countries, similarly emphasized the need for scalable, family-centered, and system-sensitive approaches (7). The practical implication is that service improvement should involve redesigning pathways as much as expanding programs (6, 8, 41, 42). Figure 2 outlines the multilevel barriers and priority support targets that shape psychosocial outcomes and access to care in children and adolescents with ASD.

## 8 Family burden and caregiver support needs

One of the clearest downstream consequences of fragmented ASD care is caregiver burden (25, 27, 29, 44–48). Families frequently bear responsibility for noticing early concerns, arranging evaluations, managing appointments, coordinating educational services, implementing strategies at home, and coping with uncertainty about the future. This work can affect caregivers’ mental health, identity, employment, social participation, finances, and overall quality of life.

Recent studies consistently document a substantial burden. Herrero et al. reported that women caregivers experienced greater objective and subjective burden and higher psychological distress than men, highlighting the gendered distribution of caregiving demands (45). Van Niekerk et al. similarly found meaningful caregiver burden among those caring for children with ASD (46). Qualitative work by Appah et al. described financial strain, emotional distress, healthcare-access difficulties, and schooling-related challenges among caregivers (42, 44, 49). D’Arcy et al. identified common themes of barriers to community engagement, financial hardship, mental health strain, and unmet support needs among caregivers of neurodiverse children (50).

The distribution and experience of caregiving may also be shaped by gendered and cultural expectations. The greater burden reported among women caregivers suggests that caregiving demands may be distributed unevenly within families (45). Evidence from racial and ethnic minority families further indicates that family and community cultural contexts, including stigma and social expectations surrounding disability, may influence caregiver well-being and experiences of support (44). Qualitative evidence from Ghana similarly suggests that stigma, social exclusion, and culturally shaped understandings of ASD may compound the emotional and social burden experienced by caregivers (49). However, generational expectations and the distribution of caregiving responsibilities among grandparents or other extended-family members were not systematically examined in the included literature. This represents an important evidence gap, particularly in settings where caregiving responsibilities may be shared across generations.

Quality-of-life research points in the same direction (29, 47, 48, 51–53). Bar et al. found that caregiver quality of life can be adversely affected in families of children with ASD and/or ADHD, with some differences across language, insurance, and racial groups (51). Davy and colleagues, in both a scoping review and a qualitative study, showed that parenting a child with ASD often restricts caregivers’ leisure, workforce participation, and community involvement, with consequences for quality of life (52, 53). In many families, the child’s needs understandably take priority over the caregiver’s own occupational participation, but this prioritization can become chronic and destabilizing without adequate support.

Family burden is intensified not only by child characteristics but also by poor system design. When referrals are delayed, recommendations are unclear, or supports are poorly coordinated, caregivers become default case managers (8, 19, 20, 23–26). This increases stress and reduces the time available for employment, relationships, self-care, and participation in ordinary family life (45, 46, 49–53). Thus, caregiver burden is not merely a family-level issue. It is also a service-quality indicator.

These findings support several practical conclusions. First, family psychoeducation and guidance should be built into care pathways routinely rather than offered only when families ask for help. Second, navigation support can reduce burden by clarifying what services exist and how to obtain them (23, 25, 26). Third, peer and parent-to-parent support may have meaningful psychosocial value. Scoping work on parent-to-parent support interventions in developmental disability contexts suggests benefits for emotional well-being, quality of life, and practical coping (54). Finally, caregiver support should include attention to mental health, not only child management skills. Parent-focused programs, including skills training and psychologically informed support, can improve caregiver well-being and family functioning (32, 50, 55).

## 9 School participation and psychosocial functioning

School is one of the most important everyday environments for children and adolescents with ASD (30, 38, 39, 56–59). It is the setting in which participation, peer relationships, communication demands, sensory experiences, emotional regulation, and academic expectations converge. It is also where support systems may either buffer difficulties or intensify them.

Recent research increasingly frames school outcomes in terms of participation rather than simple attendance or academic performance. Li et al. found that the physical environment of school was a particularly important correlate of school participation for youth with ASD, with greater difficulty in the physical environment associated with lower participation (56). This finding is clinically useful because it highlights modifiable contextual factors rather than attributing reduced participation solely to child traits. Similarly, Golos et al. reported lower participation and lower environmental support among children with ASD compared with peers, with meaningful variation across home, school, and community settings (58). Simpson et al. extended this participation-based perspective to home and community contexts, showing that sensory qualities and social demands can act as barriers and that parents often develop their own strategies to support participation (57).

These studies align with a broader neurodiversity-affirming shift toward environmental fit. Rather than asking only how the child can change, this literature asks how environments can be modified so that meaningful participation becomes possible (30, 38, 56–59). This perspective matters because traditional school-based discussions of autism often overemphasize individual deficits while underemphasizing classroom design, sensory load, routines, expectations, and peer ecology.

School systems also play a critical role in psychosocial support and transition planning (30, 31, 36, 37, 59). Hughes et al. found that although most adolescents with ASD had individualized education programs (IEPs) with transition planning, important gaps remained in living arrangement goals, school-based mental health services, and equity of planning (36). In a related population-based study, the same group reported variation and disparities in co-occurring condition identification, school-based services, and transition-related supports by sex, race or ethnicity, intellectual ability, and geographic site (37). These findings suggest that schools are not simply educational venues; they are core components of the autism support system.

For clinicians, this has two implications. First, school participation should be regarded as a health-relevant outcome because it influences emotional well-being, peer inclusion, routine, and family stress. Second, collaboration between health providers, educators, and families is essential. When healthcare recommendations are not communicated in an educationally usable way, or when schools cannot implement recommended supports, care becomes fragmented despite technically successful diagnosis (5, 30, 37, 56, 58, 59).

## 10 Adolescence, continuity of care, and transition to adulthood

Adolescence brings increasing social, academic, emotional, and practical demands, and these demands often expose the limits of fragmented care (5, 31, 36, 60, 61). For adolescents with ASD, transition challenges may include changing expectations for independence, more complex mental health presentations, planning for postsecondary education or employment, shifts in school-based entitlement, and movement from pediatric to adult-oriented healthcare systems.

Transition-related evidence suggests that planning often remains incomplete. Hughes et al. showed that while many adolescents with ASD had transition plans, disparities were evident by intellectual disability status and by the content of goals and supports (37). Other work highlights that healthcare-transition discussions frequently occur late and inconsistently (60, 61). Zhang et al. reported that only slightly more than half of adolescents and young adults with ASD in a pilot rural–urban survey had completed health-care transition, with notable variability in experiences (61). Ames et al. similarly emphasized that inclusive transition experiences require coordination across youth, caregivers, pediatric providers, and adult providers (60).

Equity concerns remain substantial in this phase as well (31, 36, 37, 60, 61). Recent work highlights that minoritized adolescents and adults with ASD face intersectional disparities in service access during transition, in part because research and service systems often undercharacterize these barriers. In practical terms, this means that transition is not only a developmental issue but also an access issue. Families who were already struggling to navigate childhood services may encounter even greater fragmentation as school-based supports decline and adult systems become more diagnosis- or eligibility-restricted.

A developmental perspective is therefore essential. Autism care should not end with early identification or be conceptualized as a childhood-limited concern. Instead, care pathways should anticipate changing needs across development, including adolescence, healthcare transfer, vocational planning, independent living supports, and sustained mental health follow-up (31, 36, 37, 60, 61). This is one of the clearest areas where service design still lags behind need.

## 11 Discussion

### 11.1 From diagnosis-centered care to developmental pathway-centered care

This narrative review suggests that the major challenges in ASD care are not limited to delayed diagnosis, insufficient service availability, or inadequate psychosocial support as separate problems. Rather, the evidence points to discontinuities across a developmental care pathway that begins with recognition of early concerns and extends through diagnostic assessment, post-diagnostic support, family adaptation, school participation, and transition to adulthood. The central contribution of this review is therefore to frame ASD care as a pathway-centered and systems-of-care issue rather than as a sequence of isolated clinical, educational, or family-level events (6, 8, 28, 31, 32).

This distinction is important because much of the existing literature examines individual components of ASD care separately. Studies of early identification emphasize diagnostic delay and disparities; service-access studies document waiting times, cost burdens, workforce shortages, and regional differences; caregiver studies describe psychological and financial burden; school participation studies focus on environmental support and participation barriers; and transition studies highlight discontinuity between pediatric, educational, and adult-oriented systems. Each of these areas provides important evidence, but when considered separately, they may understate how difficulties accumulate across development.

The revised synthesis advances understanding by connecting these domains within a developmental pathway framework. Late recognition may delay diagnostic assessment; delayed diagnosis may postpone intervention and educational planning; weak post-diagnostic coordination may increase caregiver burden; and inadequate childhood support may leave families less prepared for adolescence and transition. Timely diagnosis alone may therefore not be sufficient if diagnostic systems are not linked to accessible, coordinated, and developmentally responsive supports (6, 8, 28, 31, 32).

### 11.2 Family burden and school participation as indicators of system performance

A second interpretation emerging from the evidence is that caregiver burden and school participation should not be treated merely as secondary psychosocial outcomes. They can also be understood as indicators of how well service systems function. When assessment pathways are unclear, service eligibility is difficult to navigate, follow-up is inconsistent, and health and education systems communicate poorly, families often become the default coordinators of care (19, 20, 23–26, 45, 46, 49–53).

This shifts administrative, emotional, financial, and practical work from systems onto caregivers. In this sense, caregiver burden reflects not only the demands associated with supporting an autistic child, but also the degree to which healthcare, education, and social care systems provide or fail to provide coordinated support (29, 32, 42, 44–48, 50–53). This distinction is important because family-centered care should not become family-managed care. Families should be involved in decisions and supported in caregiving, but they should not be required to compensate for fragmented systems without adequate professional and institutional support.

School participation should be interpreted similarly. It is not simply an educational outcome or a reflection of the individual child’s functioning. School is a central environment in which communication demands, peer relationships, sensory conditions, behavioral expectations, academic requirements, and psychosocial development converge. Reduced participation may reflect poor environmental fit, inadequate accommodations, insufficient teacher training, limited collaboration between health and education professionals, or insufficient attention to sensory and social context (30, 36, 37, 56–59). Therefore, evaluation of ASD care pathways should consider not only diagnostic age or therapy receipt, but also family navigation burden, caregiver mental health, school participation, and continuity of support.

This broader interpretation of outcomes is also consistent with recent neurodiversity-affirming discussions of autistic flourishing. Care pathways should not be evaluated solely by conventional indicators such as symptom reduction, service utilization, adaptive functioning, or diagnostic timing. Although these indicators remain important for health services research, they may not fully capture whether autistic children and adolescents are supported to live meaningful, connected, and self-determined lives. The concept of autistic flourishing emphasizes fulfillment, autonomy, belonging, environmental fit, psychological well-being, and outcomes defined in relation to the lived experiences and priorities of autistic people. From this perspective, effective ASD care pathways should aim not only to reduce unmet needs and caregiver burden, but also to create conditions in which autistic children and adolescents can participate, develop, and flourish in ways that are meaningful to them (62).

### 11.3 Equity as a cross-cutting issue across the care pathway

The evidence indicates that equity should not be considered a separate or peripheral theme. Disparities appear across multiple stages of the ASD care pathway, including recognition, referral, diagnostic assessment, post-diagnostic service access, telehealth use, school-based support, and transition planning (9, 10, 12–17, 40). Socioeconomic disadvantage, race and ethnicity, language, geography, health literacy, provider availability, and service financing can shape whether children are identified, how long families wait, what services are recommended, and whether those services are usable.

This pathway perspective helps explain why improving average service capacity may not be sufficient. Expanding diagnostic services may reduce waiting times for some families but may not address language barriers, mistrust, transportation burdens, limited understanding of service systems, or uneven distribution of trained providers. Similarly, telehealth may improve access for families facing distance-related barriers, but may be less effective for families with limited digital access, limited privacy, limited caregiver time, or limited proficiency in the language used by providers (14, 40).

A critical implication is that equity-oriented ASD care requires more than adding services. It requires redesigning pathways so that they are understandable, culturally responsive, linguistically accessible, geographically reachable, and linked to practical supports. Family navigation, community-based outreach, school-health collaboration, interpreter access, targeted financial assistance, and tailored support for underserved groups may therefore be as important as increasing the number of diagnostic or therapy appointments (8, 13, 15, 23–26, 42).

### 11.4 COVID-19 and temporal heterogeneity in recent ASD service evidence

An additional consideration is that the literature included in this review was published across the pre-pandemic, pandemic, and post-pandemic recovery periods. The COVID-19 pandemic should therefore be interpreted not only as an external disruption, but also as a stress test of already fragile ASD care pathways. Pre-existing barriers, including long waiting lists, workforce shortages, weak coordination between healthcare and education, and limited family navigation support, were amplified when in-person diagnostic assessments, therapeutic services, and school-based supports were interrupted or shifted abruptly to remote formats. Evidence from the SPARK cohort indicated substantial disruptions to therapies among autistic individuals and their caregivers during the early pandemic, while subsequent analyses showed that service receipt and service recovery varied across demographic subgroups, highlighting inequities in both disruption and restoration of care (63, 64). Post-diagnostic support was also affected, as children diagnosed during the pandemic did not consistently receive all recommended services or developmental follow-up (19). These disruptions likely increased caregiver burden by requiring families to compensate for lost therapy, reduced educational supports, disrupted routines, and more complex service navigation. At the same time, the pandemic accelerated the use of telehealth and digital support models, suggesting potential routes for improving service reach while also raising equity concerns for families with limited technology access, language barriers, or lower health literacy. Accordingly, evidence from 2020 onward should be interpreted in light of this temporal heterogeneity: some barriers reflect long-standing system weaknesses, whereas others were intensified or made more visible by the pandemic.

### 11.5 Tensions, uncertainties, and gaps in the current evidence base

Several tensions and uncertainties in the current literature deserve attention. First, there is a tension between increasing identification of ASD and the capacity of systems to respond after identification. Higher prevalence estimates and improved awareness may indicate progress in recognition, but they also increase demand for diagnostic assessment, intervention, educational support, caregiver guidance, and transition planning (1, 2, 7, 8). If service systems do not expand and coordinate accordingly, earlier or more frequent identification may not translate into improved family experiences when post-diagnostic capacity remains constrained.

Second, there is a tension between family-centered care and the risk of overburdening families. Many studies appropriately emphasize caregiver involvement, family priorities, parent-mediated support, and shared decision-making. However, without adequate service coordination and structural support, family-centered care can unintentionally become family-managed care. This distinction matters because policy language that emphasizes family empowerment may obscure the practical reality that many caregivers are required to perform system-navigation work that should be supported by professionals and institutions (6, 24–26, 32, 54, 55).

Third, there is uncertainty regarding which service models are most effective across contexts. Family navigation, digital navigation, integrated primary care models, telehealth, school-based supports, parent-to-parent programs, and caregiver skills training all appear promising, but the evidence base is uneven. Many studies describe needs, barriers, feasibility, acceptability, or caregiver experience, whereas fewer compare models, examine long-term outcomes, or evaluate scalability across health systems (7, 15, 22, 49).

Transition remains a particularly underdeveloped area. Although transition planning is widely recognized as important, the literature suggests that planning is often incomplete, late, or unevenly implemented. Compared with early childhood identification and intervention, adolescence and transition to adulthood have received less integrated attention in care pathway research. The field, therefore, needs more comparative, longitudinal, and implementation-oriented evidence to determine which models work, for whom, in which settings, and through what mechanisms.

### 11.6 Implications for policy, practice, and research

Several implications follow from this critical synthesis. For healthcare systems, the priority should not be diagnosis alone, but the construction of coordinated pathways that connect recognition, referral, assessment, post-diagnostic planning, family navigation, school support, and transition preparation (5, 19–26). Diagnostic assessment should be treated as an entry point into care rather than an endpoint of evaluation.

For education systems, school participation should be treated as a core component of ASD-related support. Health and education professionals need more effective mechanisms for communication, shared planning, and translation of clinical recommendations into feasible school-based accommodations. Environmental adaptation, sensory supports, peer inclusion, and school-based mental health services should be considered part of a broader care pathway rather than separate educational concerns (36–38, 56–58).

For family support, caregiver needs should be addressed proactively. Psychoeducation, peer support, navigation assistance, culturally responsive information, and caregiver mental health support should be integrated into routine care pathways, especially for families who face socioeconomic, linguistic, or geographic barriers. Cultural beliefs, family and community understandings of autism, stigma, and experiences of discrimination may influence whether developmental concerns are discussed, whether families seek diagnostic or support services, and how an ASD diagnosis is understood, accepted, or disclosed within families and communities (13, 15, 16, 44, 49). Accordingly, efforts to reduce stigma should form part of ASD service pathways and should include culturally and linguistically appropriate education, respectful and non-blaming communication, and support that acknowledges different family understandings and concerns. Reluctance to seek help or difficulty accepting a diagnosis should therefore not be viewed simply as individual “denial,” but understood within the broader context of culture, stigma, trust, health literacy, and service accessibility. For research, future studies should move beyond documenting barriers toward evaluating solutions, including models of care coordination, family navigation, culturally responsive and stigma-reduction approaches, telehealth, school-health collaboration, and transition support (31, 36, 37, 60, 61).

### 11.7 Limitations

This review has several limitations. First, it is a narrative review rather than a systematic review. Although the search and selection procedures were made more explicit in the revised manuscript, the review did not involve formal meta-analysis, pooled effect estimation, or formal evidence grading. Screening was performed primarily by the first author, and no formal risk-of-bias instrument was applied. These methodological choices are consistent with the integrative aims of a narrative review, but they should be considered when interpreting the conclusions.

Second, the included literature was heterogeneous in design, population, setting, and outcome focus. This heterogeneity is appropriate for a review on care pathways and psychosocial support, but it limits direct comparison across studies. Evidence from qualitative studies, surveillance reports, service-access research, school participation studies, family navigation studies, and transition studies contributes different types of insight and cannot be weighted in the same way.

Third, much of the available evidence comes from high-income, English-speaking countries. Evidence from low- and middle-income countries, including rapidly developing service contexts such as China, remains comparatively limited. This geographic skew constrains the generalizability of some recommendations and highlights the need for locally grounded research on ASD care pathways across diverse health, education, and social care systems.

Fourth, because the review focused on care pathways, service access, and psychosocial support, it did not examine in detail biological mechanisms, pharmacological treatment, or intervention efficacy at the symptom level. This focus was intentional, but it means that the conclusions are most relevant to health services, family support, education, and policy rather than to biomedical treatment development.

Despite these limitations, this review suggests that the field should move from viewing ASD care as a set of discrete service episodes toward understanding it as a developmental pathway that requires continuity, coordination, and equity across systems. The most important unresolved question is not only how to identify autistic children earlier, but how to build systems that remain accessible, usable, and supportive after identification has occurred.

## 12 Conclusion

Children and adolescents with ASD require more than a timely diagnosis. They require coherent care pathways, equitable access to multidisciplinary services, family-centered support, responsive school environments, and sustained transition planning. Recent literature shows that these components remain unevenly distributed, creating avoidable burdens for children, caregivers, and systems.

For health services researchers and practitioners, the key implication is that ASD should be framed not only as a developmental diagnosis but also as a health services and care-delivery issue. Better outcomes will depend on earlier and more equitable recognition, stronger navigation and coordination mechanisms, closer integration across healthcare and education, and a developmental approach that extends beyond diagnosis into adolescence and adulthood. In practical terms, the future of autism care lies not only in identifying children earlier but also in building systems that remain usable, responsive, and supportive after identification has occurred.
