# Supplementary material for: Care pathways, service access, and psychosocial support needs in children and adolescents with autism spectrum disorder: a narrative review
Source: Front Public Health. 2026 Sep 11;14:1840171. doi: 10.3389/fpubh.2026.1840171 (PMC13614117; doi:10.3389/fpubh.2026.1840171)
Supplement: Supplementary file 1 [file Table_1.DOCX]

Supplementary Table 1. Detailed evidence map of sources used for the thematic synthesis

| **No.** | **Source** | **Year** | **Source type** | **Country/setting** | **Evidence domain** | **Key contribution to the synthesis** |
| --- | --- | --- | --- | --- | --- | --- |
| 1 | Shaw et al. | 2025 | Surveillance report | United States | Public health relevance; early identification | Provides recent ASD prevalence and early identification data from the ADDM Network, supporting the public-health importance of ASD service planning. |
| 2 | Santomauro et al. | 2025 | Global burden study | Global | Public health relevance | Provides global estimates of ASD epidemiology and burden, supporting the need for scalable and coordinated service systems. |
| 3 | Ostrowski et al. | 2024 | Public-health review | General/international | Public health relevance | Summarizes ASD etiology, epidemiology, and public-health challenges, supporting the framing of ASD as a service-planning concern. |
| 4 | American Psychiatric Association | 2022 | Diagnostic reference | International diagnostic reference | ASD definition and diagnostic framing | Provides foundational diagnostic criteria for ASD; retained as a foundational reference although not a recent empirical care-pathway study. |
| 5 | Zavaleta-Ramírez et al. | 2020 | Diagnostic pathway study | Clinical/service setting | Recognition; barriers to diagnosis | Describes pathways to ASD diagnosis and supports the framing of diagnosis as one stage within a broader care pathway. |
| 6 | Fulceri et al. | 2023 | Systematic review | International/service pathway literature | Integrated care pathways; diagnostic and post-diagnostic pathways | Synthesizes components of integrated care pathways for ASD and supports the shift from diagnosis-centered to pathway-centered care. |
| 7 | Divan et al. | 2021 | Review of reviews | Low- and middle-income countries | Service access; universal health coverage | Reviews evidence on achieving universal health coverage for young children with ASD in LMICs, supporting scalable and system-sensitive care models. |
| 8 | Malik-Soni et al. | 2022 | Health-services review | Diagnosis-to-adulthood healthcare access | Service access; lifespan continuity | Reviews healthcare access barriers for autistic individuals from diagnosis to adulthood, supporting a continuity-of-care perspective. |
| 9 | Aylward et al. | 2021 | Disparities-focused review/study | United States | Recognition; diagnostic disparities | Documents racial, ethnic, and sociodemographic disparities in ASD diagnosis. |
| 10 | Weiss et al. | 2024 | Scoping review | Youth with ASD/IDD | Recognition; diagnosis and treatment inequity | Synthesizes evidence on social inequity at the intersection of ASD, intellectual/developmental disability, diagnosis, and treatment. |
| 11 | Bent et al. | 2020 | Service pathway study | Australia | Diagnostic pathway; early diagnosis | Examines parents’ experiences of the pathway to ASD diagnosis and predictors of earlier diagnosis. |
| 12 | Miller et al. | 2023 | Population-based diagnostic pathway study | Nationally representative sample | Intersectionality; diagnostic pathway | Examines how intersectionality influences pathways to diagnosis among autistic children with and without co-occurring ADHD. |
| 13 | Onovbiona et al. | 2024 | Mixed-methods disparities study | Black families of autistic youth | Diagnostic and treatment barriers; equity | Identifies racial and practical barriers to diagnostic and treatment services for Black families of autistic youth. |
| 14 | Wagner et al. | 2025 | Telemedicine tool development study | Preschool identification context | Early identification; telemedicine; disparities | Describes a telemedicine tool designed to reduce disparities in identifying preschool children with autism. |
| 15 | Casale et al. | 2023 | Scoping review | Canada; immigrant and refugee caregivers | Service access; migration-related barriers | Synthesizes service access experiences of immigrant and refugee caregivers of autistic children. |
| 16 | Lindsay et al. | 2025 | Systematic review | Children/youth healthcare settings | Healthcare disparities; racism | Summarizes racism and racial disparities in healthcare experiences among children and youth with ASD and their caregivers. |
| 17 | Hantman et al. | 2024 | Geographic access study | United States | Service access; geographic disparities | Demonstrates geographic disparities in pediatrician availability relative to neurodevelopmental disorder prevalence. |
| 18 | Lockwood Estrin et al. | 2021 | Systematic review | Young women and girls | Barriers to diagnosis; gender | Reviews barriers to ASD diagnosis for young women and girls, highlighting gender-related diagnostic challenges. |
| 19 | Landau-Taylor et al. | 2023 | Quality-improvement study | United States | Post-diagnostic pathways; COVID-19 service access | Shows gaps in access to recommended care after ASD diagnosis during the COVID-19 period. |
| 20 | Micai et al. | 2024 | Caregiver-perspective service study | Italy | Service access; cost burden | Describes access and costs of services for autistic children and adults from carers’ perspectives. |
| 21 | Saleh et al. | 2024 | Parental satisfaction study | Saudi Arabia | Service access; service responsiveness | Examines parental satisfaction with services for persons with ASD and their families, highlighting variation in perceived service quality. |
| 22 | Pillay et al. | 2024 | Qualitative caregiver study | South Africa | Service access; waiting burden | Describes caregiver experiences of waiting, service delays, and unmet needs in ASD services. |
| 23 | Burke et al. | 2024 | Qualitative/family navigation study | Low-resourced communities | Family navigation; service access | Identifies parent perspectives needed to inform family navigator programs in low-resourced communities. |
| 24 | Burke et al. | 2024 | Digital health/service-navigation study | Family service navigation context | Digital navigation; post-diagnostic support | Describes the development of digital support for ASD service navigation based on family feedback. |
| 25 | Mazurek et al. | 2025 | Service implementation study | Underserved communities | Family navigation; primary care | Evaluates integration of family navigation into ECHO Autism for pediatric primary care in underserved communities. |
| 26 | Li et al. | 2025 | Scoping review | International/family navigation | Family navigation; diagnostic-to-intervention pathway | Synthesizes family navigation programs for children with ASD and highlights navigation as a mechanism for improving movement from screening to diagnosis and intervention. |
| 27 | Sultan | 2025 | Policy/perspective article | Child healthcare systems | Equitable service access | Discusses equitable and sustainable healthcare services for children with autism. |
| 28 | Song et al. | 2022 | Lifespan service-use study | Autism service systems | Service receipt; unmet needs; lifespan continuity | Examines service receipt and unmet needs across the autism spectrum, supporting a developmental and continuity-of-care perspective. |
| 29 | Desquenne Godfrey et al. | 2024 | Systematic review | Families of autistic children | Family functioning | Reviews family functioning in families of children on the autism spectrum and supports the family-systems dimension of the synthesis. |
| 30 | Zhao et al. | 2024 | Educational placement disparities study | China | School participation; educational equity | Provides evidence on socioeconomic disparities in educational placement for primary-school-age children with ASD in China. |
| 31 | Davis et al. | 2023 | Mental health service-use study | Transition-age autistic youth | Transition; mental health service use | Examines patterns of mental health service use during transition to adulthood. |
| 32 | Bradshaw et al. | 2022 | Clinical review | Parent-mediated autism interventions | Parent support; intervention | Summarizes advances in supporting parents in interventions for ASD and informs the caregiver support component of the review. |
| 33 | Kavanaugh et al. | 2025 | Clinical evaluation study | Clinical setting | Late diagnosis; co-occurring conditions | Examines later age of ASD diagnosis in children with multiple co-occurring psychiatric disorders. |
| 34 | Hughes et al. | 2023 | Population-based adolescent study | United States | School participation; transition planning | Examines individualized education programs and transition planning for adolescents with ASD. |
| 35 | Hughes et al. | 2024 | Population-based adolescent study | United States | Adolescence; health and education services; transition | Describes health conditions, education services, and transition planning among adolescents with ASD. |
| 36 | Waddington et al. | 2024 | Community-perspective study | Community/stakeholder setting | Support goals; participation-oriented care | Highlights community perspectives on appropriate and meaningful support goals for young autistic children. |
| 37 | Walton et al. | 2024 | Stakeholder-driven research agenda | United States | Early intervention; stakeholder roles | Highlights stakeholder priorities in autism early intervention research and service design. |
| 38 | Wallis et al. | 2024 | Telehealth disparities study | Developmental-behavioral pediatrics | Telehealth access; language disparities | Shows disparities in telehealth uptake for developmental-behavioral assessments by preferred family language. |
| 39 | Binte Mohd Ikhsan et al. | 2025 | Cross-national service access study | Europe | Service access | Provides comparative evidence on access to services for autistic people across Europe. |
| 40 | Lockwood Estrin et al. | 2023 | Caregiver perception study | New Delhi, India | Caregiver perceptions; service access | Adds non-Western evidence on caregiver perceptions of autism and neurodevelopmental disabilities. |
| 41 | Srinivasan et al. | 2021 | Survey-based needs assessment | United States; Delaware | Service access; unmet healthcare and family support needs | Documents caregiver-reported unmet healthcare and family support needs among children and youth with ASD, supporting the service access and unmet needs synthesis. |
| 42 | Emmanuel et al. | 2022 | Qualitative meta-synthesis | Racial/ethnic minority families | Family well-being; minority experience | Synthesizes well-being experiences among racial/ethnic minority family members raising a child with a neurodevelopmental disorder. |
| 43 | Herrero et al. | 2024 | Cross-sectional caregiver study | Caregiver sample | Family burden; psychological distress | Documents caregiver burden and psychological distress, including gender-related differences. |
| 44 | van Niekerk et al. | 2023 | Caregiver burden study | South Africa | Family burden | Provides evidence on caregiver burden among caregivers of children with ASD. |
| 45 | Asahar et al. | 2021 | Cross-sectional caregiver study | Malaysia | Caregiver quality of life | Examines quality of life and autism-specific child difficulties among Malaysian caregivers. |
| 46 | Robeson et al. | 2024 | Caregiver quality-of-life study | Caregivers of autistic children | Caregiver quality of life | Examines the relationship between caregiver sleep quality and quality of life. |
| 47 | Appah et al. | 2024 | Qualitative caregiver study | Ghana | Family burden; service and schooling challenges | Describes financial, emotional, healthcare-access, and schooling-related challenges among caregivers. |
| 48 | D’Arcy et al. | 2024 | Qualitative/support-needs study | Australia | Caregiver well-being; support needs | Identifies well-being and support needs of caregivers of neurodiverse children. |
| 49 | Bar et al. | 2024 | Cross-sectional quality-of-life study | United States | Caregiver quality of life | Examines quality of life among caregivers of children with ASD and/or ADHD. |
| 50 | Davy et al. | 2024 | Qualitative caregiver study | Caregiver/community participation context | Caregiver participation; quality of life | Describes restrictions in leisure, employment, community participation, and quality of life among caregivers of autistic children. |
| 51 | Davy et al. | 2022 | Scoping review | Caregiver participation literature | Caregiver quality of life; participation | Reviews caregiver leisure, employment, community participation, and quality-of-life outcomes. |
| 52 | Postma et al. | 2024 | Scoping review | Developmental disability caregiver support | Parent-to-parent support | Reviews individual parent-to-parent support interventions for caregivers of children with developmental disabilities. |
| 53 | Reichow et al. | 2024 | Systematic review/meta-analysis | Neurodevelopmental disorders | Caregiver skills training | Synthesizes caregiver skills training for caregivers of individuals with neurodevelopmental disorders. |
| 54 | Li et al. | 2024 | School participation study | Youth with ASD | School participation | Identifies youth, family, and school factors influencing school participation among autistic youths. |
| 55 | Simpson and Adams | 2024 | Parent-reported environmental study | Home/community settings | Participation; environmental support | Shows how environmental factors and parent strategies support home and community participation. |
| 56 | Golos et al. | 2024 | Participation study | Israel | Participation; environmental fit | Compares participation patterns and environmental impact among children with and without autism. |
| 57 | Hasson et al. | 2024 | Qualitative/inclusive education study | Education setting | Inclusive education; school support | Examines support experiences from parent/carer, school staff, and autistic young people’s perspectives. |
| 58 | Ames et al. | 2023 | Qualitative transition study | United States | Healthcare transition | Explores inclusion and engagement in the transition from pediatric to adult healthcare. |
| 59 | Zhang et al. | 2025 | Rural–urban transition survey | United States | Healthcare transition; rural–urban variation | Compares healthcare transition experiences among autistic adolescents and young adults in rural and urban contexts. |
| 60 | Pellicano and Heyworth | 2023 | Conceptual/theoretical article | Autistic flourishing framework | Outcome perspectives; neurodiversity-affirming care | Provides a neurodiversity-affirming framework for autistic flourishing, broadening outcome evaluation beyond symptom reduction and service use toward autonomy, belonging, fulfillment, environmental fit, and meaningful participation. |
| 61 | White et al. | 2021 | COVID-19 cohort/survey study | United States; SPARK cohort | COVID-19 service disruption; caregiver burden | Documents the impact of COVID-19 on individuals with ASD and caregivers, including disruptions to therapies and family routines during the early pandemic. |
| 62 | Tsai and Bhat | 2024 | COVID-19 service access survey analysis | United States; SPARK cohort | COVID-19 service recovery; demographic disparities | Examines demographic differences in access to health and therapeutic services during the first year of the pandemic, supporting discussion of inequities in service disruption and recovery. |
